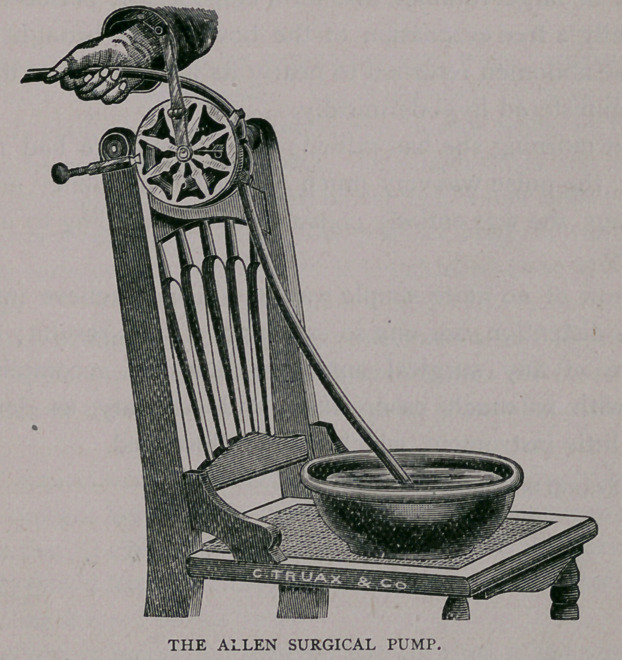# A New Use of the Allen Surgical Pump

**Published:** 1888-10

**Authors:** William Warren Potter

**Affiliations:** 284 Franklin Street; Buffalo, N. Y.


### *A NEW USE OF THE ALLEN SURGICAL PUMP.*

By WILLIAM WARREN POTTER, M. D., Buffalo, N. Y.

The following case will serve to indicate a somewhat novel way in which the Allen Surgical Pump may be employed with advantage:

THE ALLEN SURGICAL PUMP.

On the evening of the fifteenth day of June, 1888, I was called to visit Mrs. X. as a consultant. She was found to be suffering with peritonitis, probably of a puerperal origin, and seemed to be *in extremis.* Her pulse was 140; temperature, ninety-seven and a-half; surface bathed with clammy perspiration; breathing short and hurried, and she was suffering great pain withal. There was great distention of the abdomen from flatus, which had resisted all methods to effect its relief, among which were repeated attempts at rectal catheterization, warm water injections, etc. An effort was made to pass a soft rubber rectal tube, which met with opposition after it had passed the sphincter less than three inches. It was immediately coupled to the pump, and water at 110 degrees F. pumped into the bowel by that instrument. The tube advanced a little way, then stopped, more water was pumped in, when it again advanced three or four inches and again stopped; whereupon, the pump was again turned and the tube again advanced, and in this way it was finally passed its whole length, two feet six inches, into the intestinal canal. It was uncoupled, the water flowed out, and immediately the flatus escaped in large volumes, and for a considerable period of time.

Finally a free evacuation of the bowels was brought about, when the abdomen returned to nearly its normal girth; morphia was administered hypodermically.

Next morning she had rallied; the temperature had risen to normal; the pulse was very much reduced in frequency, and after some days she was entirely convalescent, proceeding to ultimate recovery.

I know of no more simple way in which to relieve intestinal gaseous distention, nor one so satisfactory in its results; nor am I aware of any surgical appliance that will accomplish the result with so much ease, safety, and certainty, as does this unique little instrument, which is here illustrated.

284 FRANKLIN STREET.

## Figures and Tables

**Figure f1:**